# Tissue specific diversification, virulence and immune response to *Mycobacterium bovis* BCG in a patient with an IFN-γ R1 deficiency

**DOI:** 10.1080/21505594.2020.1848108

**Published:** 2020-12-24

**Authors:** Cecilia B. Korol, Shamira J. Shallom, Kriti Arora, Helena I. Boshoff, Alexandra F. Freeman, Alejandra King, Sonia Agrawal, Sean C. Daugherty, Timothy Jancel, Juraj Kabat, Sundar Ganesan, Marina N. Torrero, Elizabeth P. Sampaio, Clifton Barry, Steve M Holland, Hervé Tettelin, Sergio D Rosenzweig, Adrian M. Zelazny

**Affiliations:** a Department of Laboratory Medicine, Clinical Center, NIH, Bethesda, USA; b Laboratory of Clinical Immunology and Microbiology, National Institute of Allergy and Infectious Diseases, NIH, Bethesda, USA; c Department of Pediatric Immunology, Hospital Luis Calvo MacKenna, Universidad De, Chile, Chile; d Department of Microbiology and Immunology, Institute for Genome Sciences, University of Maryland School of Medicine, Baltimore, USA; e Department of Pharmacy, Clinical Center, NIH, Bethesda, USA; f Department Biological Imaging Section, Research Technologies Branch, National Institute of Allergy and Infectious Diseases, NIH, Bethesda, USA

**Keywords:** Tuberculosis, BCG, antibiotic resistance, immunodeficiency, Mendelian susceptibility to mycobacterial diseases, interferon-γ

## Abstract

**Summary**: We characterized *Mycobacterium bovis* BCG isolates found in lung and brain samples from a previously vaccinated patient with IFNγR1 deficiency. The isolates collected displayed distinct genomic and phenotypic features consistent with host adaptation and associated changes in antibiotic susceptibility and virulence traits.

**Background**: We report a case of a patient with partial recessive IFNγR1 deficiency who developed disseminated BCG infection after neonatal vaccination (BCG-vaccine). Distinct *M. bovis* BCG-vaccine derived clinical strains were recovered from the patient’s lungs and brain.

**Methods**: BCG strains were phenotypically (growth, antibiotic susceptibility, lipid) and genetically (whole genome sequencing) characterized. Mycobacteria cell infection models were used to assess apoptosis, necrosis, cytokine release, autophagy, and JAK-STAT signaling.

**Results**: Clinical isolates BCG-brain and BCG-lung showed distinct Rv0667 *rpoB* mutations conferring high- and low-level rifampin resistance; the latter displayed clofazimine resistance through Rv0678 gene (MarR-like transcriptional regulator) mutations. BCG-brain and BCG-lung showed mutations in *fadA2, fadE5, and mymA* operon genes, respectively. Lipid profiles revealed reduced levels of PDIM in BCG-brain and BCG-lung and increased TAGs and Mycolic acid components in BCG-lung, compared to parent BCG-vaccine. *In vitro* infected cells showed that the BCG-lung induced a higher cytokine release, necrosis, and cell-associated bacterial load effect when compared to BCG-brain; conversely, both strains inhibited apoptosis and altered JAK-STAT signaling.

**Conclusions**: During a chronic-disseminated BCG infection, BCG strains can evolve independently at different sites likely due to particular microenvironment features leading to differential antibiotic resistance, virulence traits resulting in dissimilar responses in different host tissues.

## Introduction

Almost 88% of the world population gets vaccinated with the *Bacille Calmette–Guérin* (BCG) vaccine during the first month of life [1] aiming for protection against severe *Mycobacterium tuberculosis* infection. Inoculation of live BCG-vaccine is harmless in most children; however, life-threatening complications following BCG vaccination are well documented in children with both primary and acquired immunodeficiencies [1]. Defects affecting IL-12 and IFN-γ signaling can lead to fatal, chronic, localized, or disseminated BCG infections [2,3] in these individuals upon vaccination. From a mycobacterial perspective, many of the mycobacterial virulence genes encode for enzymes in lipid metabolism pathways, cell surface proteins, regulators, and proteins of signal transduction systems or factors enhancing survival inside the host macrophages [4].

*M. tuberculosis* and attenuated BCG have numerous consequences for host–pathogen interaction and vaccine efficacy. It is likely that the RD1 deletion in attenuation of BCG and the resulting loss of ESX-1 functions has occurred during the long-term in vitro passage that occurred in the original virulent *M. bovis* [5]. Studying within-host genetic diversity of BCG in a patient may identify adaptations to antibiotic exposure and immune response. Although still a matter of debate, one strategy involves skewing the equilibrium toward necrosis rather than apoptosis, promoting the release of bacilli for subsequent infection of the surrounding cells [3,6,7]. In contrast, death of the infected cells by apoptosis triggers the recruitment of professional antigen-presenting cells that involve the adaptive immune response, which clearly is disadvantageous for mycobacterial survival [7]. Modulation of host cell cytokine production during infection can also contribute to mycobacterial dissemination or inhibition of pathogen autophagy [8,9]. Autophagy is a physiologic process that eliminates intracellular components such as damaged organelles, cell components, or proteins and also has a key role in the elimination of intracellular pathogens such as mycobacteria [9,10]. Pathogenic mycobacteria have been described to down-regulate autophagy [9,10]. Another mechanism to control macrophage growth of intracellular pathogens is the activation through IFNγ-inducible genes and activation of Janus Kinase-signal transducer and activator of transcription (JAK-STAT) pathway. Pathogenic mycobacteria can alter STAT signaling either by decreasing IFNγ receptor levels, association with CREB (a transcriptional co-activator), or up-regulation of the inhibitors of the SOCS family [11]. Accordingly, host genetic defects in the IL-12/IFNγ pathway are linked to increased susceptibility to mycobacterial disease [11,12].

Herein we report a patient previously reported as case VII.1 in Sologuren I. *et al*. [13] and patient 3 in Bax HI *et al*. [14] with partial recessive IFNγR1 deficiency who developed disseminated BCG infection after neonatal vaccination with BCG-vaccine. Different BCG isolates; BCG-brain was recovered from his scalp wounds and BCG-lung from lungs, both of which were derived from the original vaccine strain (BCG-vaccine Danish Strain 1331) from Chile (Statens Serum Institute, Denmark). This study allowed us to determine distinctive genotypic and phenotypic characteristics of these strains in distinct sites of infection, which in turn resulted in differences affecting their growth rate, antibiotic susceptibility, virulence traits, and the microbial interaction with the host’s inflammatory and immune responses.

## Methods

### Patient’s case

The patient previously reported as case VII.1 in Sologuren I. *et al*. [13] and patient 3 in Bax HI *et al*. [14] presented to the National Institutes of Health (NIH) at the age of 3 y with a disseminated BCG infection. He received BCG-vaccine in Chile shortly after birth and presented with left axillary lymphadenitis at 2 months of age, which was culture positive for *M. bovis* BCG. He was initially treated with isoniazid (INH) monotherapy with poor tolerance and response. By 2 y of age, he had developed fevers, failure to thrive, hydrocephalus, developmental delay, and granulomatous osteolytic bone lesions in multiple ribs and the right femur. Immunologic and genetic studies led to the diagnosis of partial recessive IFNγR1 deficiency (homozygous p.I87T). His anti-mycobacterial treatment was modified to include isoniazid (INH), ethambutol (ETH), rifampin (RIF), and streptomycin. IFNγ was added to the regimen but was rapidly discontinued because of further neurologic decline and poor tolerance.

Upon arrival to NIH at age 3 y his anti-mycobacterial regimen consisted of INH, clarithromycin, and RIF (table 1). He had hydrocephalus with exophthalmos and developmental delay. A chronic draining lesion was present on the right temporal region of his skull, where a prior brain biopsy was performed and had yielded mycobacteria. An occipital draining lesion persisted at another brain biopsy site. Brain imaging showed multiple enhancing lesions and enlarged asymmetric ventricles [14]. Chest imaging revealed a pleural-based mass on the right lung and bilateral hilar adenopathy. Splenomegaly, retroperitoneal adenopathy, and healing bony lesions of the right femur and scoliosis were also observed.

**Table 1.** Patient treatment and timeline of chronic BCG-osis at NIH

Age (y)	Culture site	Smear	Culture	Imaging-brain	Imaging-lung	Antibiotics	Cytokine adjuvants
3	Scalp	Many	Positive	Multiple enhancing lesions	Pleural mass	Clari, INH, RIF	None
3	Lung	Many	Positive	Multiple enhancing lesions	Pleural mass	Clari, INH, RIF	None
3.2	Scalp	Negative	Positive	Stable	Stable	Clari, INH, RIF, ETH, AMI, Levo	IFNγ
3.3	Scalp	Few	Negative	Stable	Increased size	Azi, INH, RIF, ETH,AMI	IFNγ
3.3	Lung	Moderate	Liquid media only	Stable	Increased size	Azi, INH, RIF, ETH,AMI	IFNγ
3.7	Scalp	Negative	Negative	Stable	Increased size	Azi, INH, ETH, Levo, LZD, Clofaz, Capreo	IFNγ and IFNα
3.8	Scalp	Negative	Negative	Stable	Stable	Azi, INH, ETH, Levo, LZD, Clofaz, Capreo	IFNγ and IFNα
4.0	Lung	Negative	Negative	Stable	Increased size	Azi, INH, ETH, Levo, LZD, Clofaz	IFNγ and IFNα

Clari, clarithromycin; INH, isoniazid; RIF, rifampin; ETH, ethambutol; AMI, amikacin; Levo, levofloxacin; Azi, azithromycin; LZD, linezolid; Clofaz, clofazimine; Capreo, capreomycin.

The scalp wounds result from brain biopsies that were chronically draining upon his presentation. When the antibiotics were broadened and Interferon-gamma and Interferon-alpha were started, the scalp lesions improved. However, there was worsening of a pleural-based lung lesion, which was labeled “lung,” leading to the lung biopsy cultures and the clinical impression of the lung disease worsening while the brain/scalp lesions improved. The brain imaging findings remained stable. Only certain time points were possible for the cultures labeled as “lung” due to this being an intra-thoracic biopsy interventional radiology requiring anesthesia support due to his young age. The scalp lesions were initially easy to culture since they were external, but cultures then became negative fairly shortly after broadening antibiotics.

Cultures obtained from the temporal draining site as well as a lung biopsy of the dense infiltrate were smear and culture positive for *M. tuberculosis* complex, consistent with *M. bovis* BCG. His anti-mycobacterial regimen was expanded to include ETH, levofloxacin, linezolid, clofazimine, capreomycin, and azithromycin, in addition to INH, and RIF. Further, adjuvant treatment with IFNα and IFNγ were added, sequentially with close monitoring and were tolerated (table 1) [14]. Soon after, the draining lesions on his scalp improved and he had a better clinical condition with weight gain and normalization of laboratories. However, the lung mass continued to worsen. With persistent therapy, improvement of the lung mass was achieved 15 months after the combined antibiotics and cytokine adjuvants, achieving complete resolution over the following 6 months (table 1).

Drug dosing was based on recommendations and published experience in pediatric patients with disseminated *M. bovis* BCG or extra-pulmonary *M. tuberculosis*. Therapeutic drug monitoring (National Jewish Health Pharmacokinetics Laboratory, Denver, CO) was used to ensure adequate drug exposure and to guide any dose adjustments during treatment. Serum concentrations were collected at multiple time points for azithromycin, capreomycin, clarithromycin, clofazimine, ETH, INH, levofloxacin, linezolid, and RIF; however, no cerebrospinal fluid (CSF) concentrations were collected. RIF serum concentrations were highly variable and ranged from 1.2 to 8.5 mcg/ml. The patient’s guardians provided informed consent on approved protocols of the NIH.

### Microbiology

**Figure 1.**
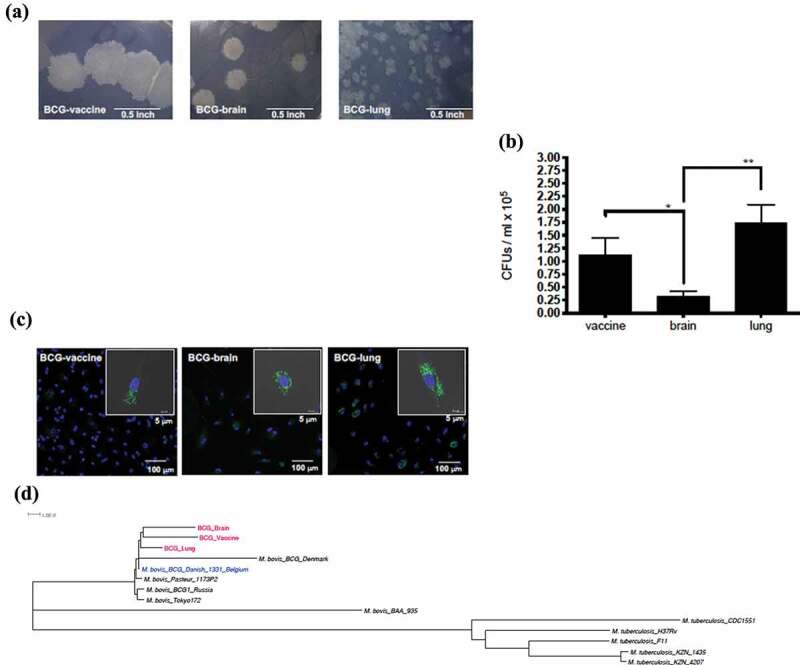
Evaluation of mycobacterial Isolates: (a) Difference in colony morphology of *Mycobacterium bovis* isolates within the BCG vaccinated patient with IFNγR1 deficiency compared to the vaccine *Mycobacterium bovis* BCG isolate. Colonies of the BCG strains at 3 weeks growth in Middlebrook 7H11 plate (100× magnification, Bar = 0.5 inch). (b) Quantification of colony-forming units (CFUs); from the lysate of peripheral mononuclear cells at d 2 of infection by the 3 strains. Data, mean ± SE (n = 6, **p < 0.01; *p < 0.05; two-tailed paired Student’s *t*-test). (c) Monocyte-derived macrophages were infected with GFP-transfected strain; at MOI of 2 for 2 d. Slides were made with DAPI staining for nucleic acid content. Representative infected cell of each strain, with also gray color as visible light showing bacteria inside of the limits of the cell (bar = 5 μm), with field showing more cells on smaller scale (bar = 100 μm). (d) Core genome phylogenetic analysis of 14 genomes; 3 BCG strains (BCG-vaccine, BCG-brain, and BCG-lung), 5 representative genomes of *M. tuberculosis* (H37Rv, CDC1551, F11, KZN 1435, and KZN 4207), and 6 *M. bovis* isolates (BAA_935, Tokyo 172, BCG1 Russia, BCG Pasteur 1173P2, BCG Danish 1331 Belgium, and BCG Denmark)

Mycobacteria were grown on Middlebrook 7H11 agar (Remel, Lenexa, KS) for 2–3 weeks. Strains were cultured to late exponential phase in Middlebrook 7H9 broth supplemented with albumin, dextrose, and catalase (ADC) and tween-80 (0.05%), washed twice with PBS, frozen, and stored at −70°C until use. Colony-forming units (CFUs)/ml were assessed by plating serial dilutions on Middlebrook 7H11 agar (figure 1a).

The BCG-vaccine Danish Strain 1331 (BCG-vaccine) from Chile (Statens Serum Institute, Denmark) and the NIH BCG-brain and BCG-lung clinical isolates were confirmed as *M. tuberculosis* complex by Maldi-TOF-MS and antibiotic susceptibility was performed. Antibiotic susceptibility testing of clinical and vaccine strains was performed as described previously (table 2) [15].

**Table 2.** Antibiotic MIC values of vaccine, lung, and brain *Mycobacterium bovis* BCG strains at 14 d. Values in bold represent MIC values associated with mutations as discussed in the text

MIC (µg/ml)	BCG-Vaccine	BCG-Brain	BCG-Lung
Streptomycin	0.39	0.19	0.19
Kanamycin	1.5	0.78	0.78
Amikacin	0.19	0.19	0.09
Ethambutol	1.5	1.5	1.5
Isoniazid	0.19	0.04	0.09
Rifampin	<0.001	**>=1.2**	**0.14**
Rifapentine	<0.001	>=0.1	0.06
Capreomycin	0.78	0.39	0.39
Levofloxacin	0.19	0.19	0.19
Moxifloxacin	0.09	0.04	0.04
Ciprofloxacin	0.31	0.31	0.15
Clofazimine	0.19	0.78	**3.12**
Ethionamide	25	12.5	50
Linezolid	0.39	0.78	0.39
Clarithromycin	<0.02	0.19	0.04
Azithromycin	0.78	1.5	0.39

### Assays for cell association studies

For assays with peripheral blood mononuclear cells (PBMCs), blood samples from healthy volunteers were obtained under appropriate protocols through the Department of Transfusion Medicine, NIH. PBMCs were obtained by a Ficoll gradient (Lonza Group, Basel, Switzerland). Cells (10^5^) were plated on a 96-well plate with infection media and infected for 2 d (MOI = 2) for assessment of cell-associated mycobacteria (figure 1b). PBMCs were infected with the 3 BCG strains and assessed for cell-associated mycobacteria.

For confocal microscopy, BCG-vaccine, BCG-lung, and BCG-brain strains were transformed with the green fluorescent protein-expressing pMSP12:GFP plasmid grown and prepared as described above using media supplemented with kanamycin 25ug/ml (figure 1c). For experiments assessing CFUs of cell-associated mycobacteria, cells were washed twice with PBS and then lysed with Triton-X100 1% (Sigma-Aldrich, St. Louis, MO) in PBS. CFUs were assessed by quantitative plating.

### BCG genomes sequencing and analysis

The draft genomes of the BCG-vaccine, the BCG-lung, and the BCG-brain strains were each sequenced on a full run of 150 nucleotide paired-end reads on an Illumina MiSeq instrument. Reads were trimmed to remove Illumina adaptor sequences as well as to remove low-quality bases using Trimmomatic [16]. Read coverage was then sub-sampled randomly on a range from 80x to 180x using fastqSample; a utility within the Celera Assembler package [17] followed by de novo assembly using MaSuRCA v2.2.1 [18]. The most optimal assemblies were obtained with a k-mer size of 49 and coverages as follows: 80x coverage with 244 contigs and a contig N50 of 39,047 (BCG-vaccine), 180x coverage with 239 contigs and N50 of 42,957 (BCG-lung), and 140x coverage with 253 contigs and N50 of 46,664 (BCG-brain). Contigs were annotated using the Institute for Genome Sciences (IGS) Annotation Engine and the genomes of the 3 strains were compared as previously described [19]. Sequenced and annotated draft genomes reported here are publicly available at GenBank with accession numbers: MQTT00000000 (BCG-brain), MQTU00000000 (BCG-lung), and MQTV00000000 (BCG-vaccine).

Whole-genome multiple sequence alignments were performed with Mugsy software within the CloVR infrastructure using the CloVR Comparative pipeline [20]. Core segments including single nucleotide polymorphisms (SNPs) were analyzed with Phylomark software [21]. Genes harboring mutations leading to non-synonymous amino acid changes, premature stop-codons, or frame-shifts were identified. The alignment and genome annotation were used to generate Mugsy clusters of syntenic orthologs Comparative genomic data were interrogated using the Sybil platform [22].

High-quality SNPs supported by deep Illumina read coverage among our 3 BCG strains were identified by mapping the reads to the closely related gap-free (finished) genome BCG Danish 1331 Belgium [NZ CP039850]. Mapping was performed with bwa mem with default parameters [23]. SAM files were processed using samtools view, sort, and index [24]. The sorted BAM files were then subjected to bcftools mpileup and call to generate VCF files (bcftools mpileup -f sorted.bam | bcftools call – ploidy 1 -mv -Ov -o calls.vcf) [25]. All SNPs with QUAL≥100 were viewed in IGV [26] and those in coding regions were inspected to determine if they were synonymous or non-synonymous. In addition, SNPs were annotated using snpEff 4.3 t (build 2017–11-24 10:18) [27] using parameters -ud 0 (no annotation upstream and downstream of SNPs) and -noLof (no eukaryotic loss-of-function and nonsense-mediated decay annotations) to reveal complete details on the effect of SNPs on bacterial genes (Supplementary file 1). We validated the SNPs in coding regions between the BCG-vaccine, BCG-brain, and BCG-lung genomes by Sanger sequencing and IGV [26] as described in table 3.

**Table 3.** Identification of mutations in the clinical isolates that have *M. tuberculosis* H37Rv homologs

H37Rv gene product name H37Rv locus tag	Function H37Rv locus tag	Gene product name BCG Danish 1331	BCG Danish 1331 locus tag and SNP position in genome	BCG-vaccine	BCG-brain	BCG-lung
Acetyl-CoA acetyltransferase *fadA2* Rv0243	Lipid degradation	Acetyl-CoA C-acetyltransferase	FCU26_RS01340 291,101	BIS44_3264 Wild type	BIT18_1027 568A>C Thr190Pro	BIT17_0787 Wild type
Acyl-CoA dehydrogenase Rv0244c (probable *fadE5*)	Lipid degradation	Butyryl-CoA dehydrogenase	FCU26_RS01345 293,963	BIS44_3263 Wild Type	BIT18_1028 35 T > G Val12Gly	BIT17_0786 Wild type
Diacylglycerol *O*-acyltransferase Rv3087	Mycolic acid synthesis, virulence, lipid metabolism	Wax ester/triacylglycerol synthase family *O*-acyltransferase	FCU26_RS16610 3,387,640	BIS44_3892 Wild type	BIT18_3976 Wild type	BIT17_3018 413A>G His138Arg
RNA polymerase, beta subunit, *rpoB* Rv0667	RNA synthesis, Rifampin resistance	DNA-directed RNA polymerase subunit beta	FCU26_RS03640 Brain SNP 762,861 Lung SNP 762,912	BIS44_1904 Wild type	BIT18_3441 1304A>T Asp435Val	BIT17_4071 1355 T > C Leu452Pro
Heat-shock protein transcriptional repressor *HrcA* Rv2374c	Virulence, detoxification, adaptation	Heat-inducible transcriptional repressor	FCU26_RS12720 2,599,334	BIS44_2080 Wild Type	BIT18_1210 Wild Type	BIT17_4383 881 G > T Gly294Val
Probable conserved transmembrane transport protein MmpL5 Rv0676c	Fatty acid transport	Siderophore RND transporter MmpL5	FCU26_RS03690 777,504	BIS44_1915 Wild type	BIT18_3430 2728A>C Ile910Leu	BIT17_4082 Wild type
Hypothetical protein Rv0678	Putative Regulatory function, Clofazimine resistance	Hypothetical protein	FCU26_RS03700 781,024	BIS44_1917 Wild type	BIT18_3428 Wild type	BIT17_4084 BIT17_4085 (284 T > A) Leu95* Point mutation (amber)
Amidophosphoribosyltransferase *purF* Rv0808	*De novo* purine biosynthesis	Amidophosphoribosyltransferase	FCU26_RS04420 904,098	BIS44_0866 Wild type	BIT18_0945 Wild type	BIT17_1246 1235 G > A Arg412His
Hypothetical protein Rv0877	Unknown	DUF3027 domain-containing protein	FCU26_RS04830 977,277	BIS44_2274 Wild type	BIT18_0488 BIT18_0489 Frameshift	BIT17_3190 Wild type
Hypothetical protein Rv2164c	Unknown	Hypothetical protein	FCU26_RS11595 2,387,417	BIS44_0261 Wild type	BIT18_0263 Wild type	BIT17_3438 695A>G Glu232Gly
Hypothetical protein Rv2267c	Unknown	Sulfotransferase	FCU26_RS12145 2,500,583	BIS44_2709 779A>G His260Arg	BIT18_3241 779A>G His260Arg	BIT17_4495 779A>G His260Arg
PPE family protein PPE54 Rv3343c	Unknown	PPE family protein	FCU26_RS18410 3,745,609	BIS44_1763 1301 C > A Ser434Tyr	BIT18_2846 1301 C > A Ser434Tyr	BIT17_2415 1301 C > A Ser434Tyr
Acyl-CoA dehydrogenase *fadE34* Rv3573c	Lipid degradation	Acyl-CoA dehydrogenase	FCU26_RS19620 4,016,308	BIS44_3539 Wild type	BIT18_0278 1513 T > G Trp505Gly	BIT17_0253 Wild type
Hypoxanthine phosphoribosyltransferase *hpt* Rv3624c	Salvage of purines	Hypoxanthine phosphoribosyltransferase	FCU26_RS19850 4,059,295	BIS44_1560 Wild type	BIT18_0654 347 T > G Val116Gly	BIT17_3703 Wild type

A neighbor-joining phylogenetic tree of 14 representative genomes – the 3 BCG strains (BCG-vaccine, BCG-brain, and BCG-lung), 5 *M. tuberculosis* genomes (H37Rv, CDC1551, F11, KZN 1435, and KZN 4207), and 6 *M. bovis* isolates (BAA_935, Tokyo 172, BCG1 Russia, BCG Pasteur 1173P2, BCG Danish 1331 Belgium, and BCG Denmark) was built using MEGAX [28] and visualized with FigTree (figure 1d) (http://tree.bio.ed.ac.uk/software/figtree) [29].

### Lipid analysis

**Figure 2.**
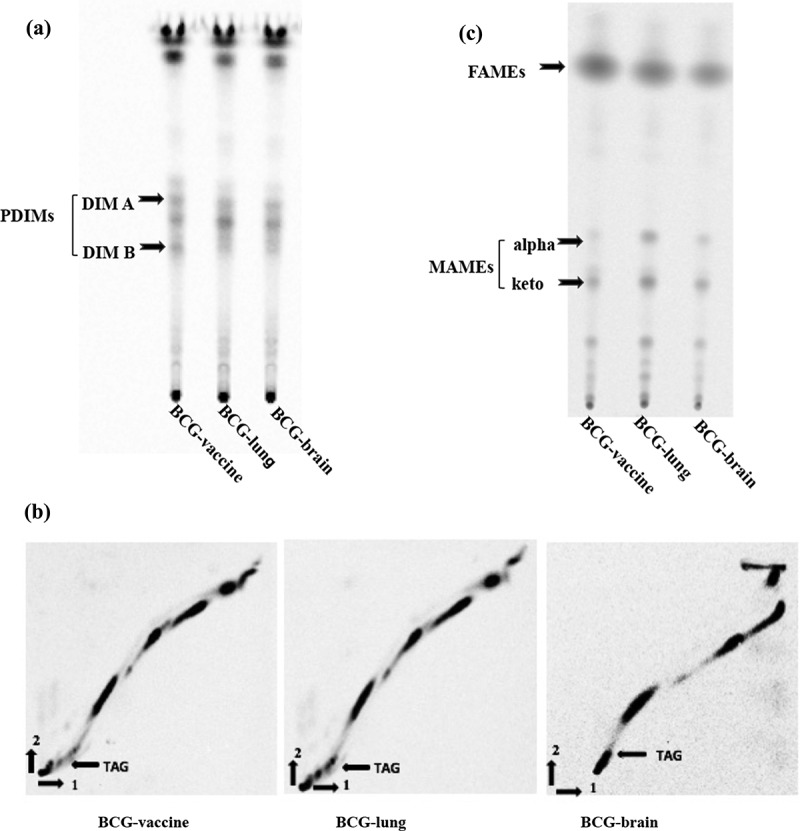
*Mycobacterium bovis* lipid profiles. TLC images of BCG-vaccine and clinical isolates from the BCG vaccinated patient with IFNγR1 deficiency are shown

Mycobacterial lipids: phthiocerol dimycocerosates (PDIMs), phenolic glycolipids (PGLs), triacylglycerols (TAGs), fatty acid methyl esters (FAMEs) and mycolic acid methyl esters (MAMEs) were separated on TLC plates as previously described [30–32]. For PDIMs, PGLs and TAGs, 50 µl [^14^C]-propionate (54 mCi/mmol, 0.1 µCi/µl; 5 µCi total) was added to bacteria (50 ml) in logarithmic growth and incubated for 48 hours at 37ºC (figures 2a and 2b). Bacterial pellet was re-suspended in Methanol/0.3% NaCl (10:1) followed by two extractions with petroleum ether. The samples were dried by evaporation, re-suspended in 300 µl chloroform and radioactive counts quantified. Equivalent sample amounts, normalized for radiation counts were loaded on silica gel G60 plates (EMD Millipore, Billerica, MA) TLC plates. The TLC plates were run using petroleum ether: diethylether (90:10, v/v) [30], and PDIM lipids were visualized after 7 d of exposure to a phosphor screen using a PhosphorImager system (Molecular Dynamics, Sunnyvale, CA, USA). Extracts were obtained in two biological replicates with two technical replicates. The same lipid extract was used to separate the triacylglycerols (TAGs) using the solvent petroleum ether-ethyl acetate (98:2, v/v, 3 runs) in the first dimension, rotated 90° and run in petroleum ether: acetone (98:2, v/v, 1 run) in the second dimension [31]. Samples were run on separate silica G60 TLC plates and TAGs [33] were visualized after 18 d of exposure.

(A) PDIMs (Phthiocerol dimycocerosates): [^14^C]-propionate radiolabeled lipids from BCG-vaccine, BCG-brain, and BCG-lung were dissolved in chloroform and loaded onto a Silica G60 TLC plate, run in petroleum ether:diethylether (90:10, v/v). The positions of the DIM A and DIM B (arrows) are indicated. (B) Triacyglycerol (TAG): [^14^C]-propionate radiolabeled TAGs from BCG-vaccine, BCG-brain, and BCG-lung were dissolved in chloroform and loaded onto a Silica G60 TLC plate. TAGs were separated in the first direction with petroleum ether/ethyl acetate (98:2, v/v, 3 runs) and rotated 90° and separated using petroleum ether:acetone (98:2, v/v, 1 run) in the second direction. TAGs position (arrows) is indicated for the different extracts. (C) Mycolic acids: [^14^C]-acetate radiolabeled Mycolic acids from BCG-vaccine, BCG-brain, and BCG-lung were dissolved in dichloromethane and loaded on a silica gel F254 thin-layer chromatography (TLC) plate run in hexane:ethyl acetate (19:1, v/v, two runs). *M. bovis* BCG-derived isolates have the FAMEs and alpha and keto bands which constitute the MAMEs are indicated with arrows. *M. bovis* BCG isolates lack the methoxy band which is present in *M. tuberculosis* isolates. Radiolabeled lipids were visualized using a PhosphorImager system.

For FAMEs and MAMEs (figure 2c), 50 µl [^14^C]-acetate (56 mCi/mmol, 1 µCi/ml; 50 µCi total) was added to bacteria in logarithmic growth and incubated for 8 hours at 37ºC. The ^14^C-labeled cells were harvested and washed with PBS. The lipids from cell pellets were hydrolyzed with 2 ml of 20% tetra-n-butylammonium hydroxide (TBAH) overnight at 100°C. Fatty acids were esterified by adding 1 ml of CH_2_Cl_2_ and 300 µl CH_3_I at room temperature for 1 h. The lower phase was dried and re-suspended in 3 ml of di-ethyl ether, and after centrifugation, the organic phase was dried, and lipids were re-suspended in 300 μl dichloromethane. Equivalent sample amounts, normalized for radiation counts were loaded on a silica gel 60 F254 thin-layer chromatography (TLC) plate (EMD Millipore, Billerica, MA) and resolved using hexane/ethyl acetate (19:1, v/v, 2 runs) [32]. Lipids were visualized by using a PhosphorImager system after 7 d of exposure (Molecular Dynamics, Sunnyvale, CA). Extracts were obtained from two biological replicates with technical replicates. Images of these lipids were quantified using an open-source software ImageJ from NIH (https://imagej.nih.gov/ij/). The values were compared to BCG-vaccine isolate for statistical significance using one-way ANOVA with Bonferroni’s multiple comparisons test in GraphPad Prism (San Diego, CA).

For Nile Red staining of neutral lipids (which include abundant TAGs) mycobacteria were grown to an OD_650_ of 0.2–0.3 and 1 ml cells were pelleted from each culture. The cell pellets were re-suspended in 1 ml of 1x PBS and Nile Red dye (Sigma, N3013-100 MG, 0.5 mg/ml stock concentration in ethanol) was added to the cell suspension at a final concentration of 10 µg/ml. The cells were incubated at 37°C for 1 hr in presence of the dye. After incubation, the cells were pelleted by centrifugation at 13,000 rpm for 10 min. The pellets were washed 3 times with 1x PBS and then resuspended in 400 µl of 100% ethanol. The samples were then incubated at 37°C with shaking for 15 min to allow for dye extraction to occur. The cells were pelleted once again, and supernatant was collected. The supernatant was read in triplicate wells of a 96-well black solid bottom plate (Corning 3916) at Excitation = 544 nm and Emission = 590 nm using Fluostar Optima (BMG Labtech) plate reader. The assay was performed with three biological replicates and technical replicates [34]. The values were compared to BCG-vaccine isolate for statistical significance using one-way ANOVA with Bonferroni’s multiple comparisons test in GraphPad Prism (San Diego, CA).

### Assays with monocyte-derived macrophages

Monocyte-derived macrophages (MDM) were differentiated from healthy donor monocytes following negative selection of CD14+ cells from PBMCs (EasySep Human Monocyte Enrichment Kit, STEMCELL Technologies, Seattle, WA) and 5 d incubation with growth media supplemented with 100 ng/ml M-CSF (PeproTech, Rocky Hill, NJ). Following replacement with infection media, GFP-transfected BCG strains (figure 1c) were added for 2 d (MOI = 0.05–2) and cells washed and fixed in paraformaldehyde 4% (USB, Cleveland, OH). Slides were then permeabilized with Triton-X100 1%, blocked with Triton-X100 1% and 10% FBS, and incubated with primary antibody LC3B (Abgent, San Diego, CA), goat anti-Rabbit IgG (H + L) secondary antibody, Alexa Fluor 647 conjugate (Thermo Fisher Scientific, Waltham, MA) and then stained with DAPI (Cell Signaling Technology, Danvers, MA). Quantification of nuclei and LC3B-puncta was performed with Imaris 8.1 software spot-counting feature (Supplementary Figure 2c) (Bitplane AG, Zurich, Switzerland).

Immunoblots were performed on protein lysates with Mcl-1, β-actin (figure 4) (Enzo Life Sciences, Inc., Farmingdale, NY), STAT1 (BD Biosciences, San Jose, CA), pSTAT3 (Y705, Santa Cruz Biotechnology, Santa Cruz, CA), Sequestosome-1 (Bethyl Laboratories, Inc., Montgomery, TX) and from Cell Signaling Technology (Danvers, MA): SOCS3, STAT1, pSTAT1 (Y701), pSTAT4 (Y693), pSTAT5 (Y694), STAT5, LC3B and SQSMT1 antibodies following manufacturers’ recommendations (figure 6, and Supplementary Figure 2). All statistical analyses related to immunoblots were done using one-way ANOVA with Bonferroni’s multiple comparisons test in GraphPad Prism (San Diego, CA).

### Mycobacteria–human cell studies

Human monocytic THP1 cells were differentiated and then infected with BCG-vaccine, BCG-brain, or BCG-lung strains. Supernatants were assessed for cytokine levels (IL-1β, IL-18, IL-6, IL-10, TNF⍺, and GM-CSF) (figure 5a-F). Human monocytic THP1 cells were maintained in RPMI 1640 with 10% fetal bovine serum (FBS), penicillin 100 U/mL, streptomycin 100 µg/mL, and L-glutamine 2 mM (Gibco BRL, Gaithersburg, MD), referred as “growth media.” Cells (10^5^) were seeded in a 24-well plate and stimulated overnight with 25 nM Phorbol-12-Myristate-13-Acetate (Sigma-Aldrich, St. Louis, MO) to obtain differentiated THP1 cells (dTHP1). dTHP1 cells were then washed with PBS (K·D Medical, Columbia, MD) and growth medium was added. After 2 d, growth medium was replaced by “infection medium” (growth media without streptomycin) and cells were infected for 2 d with BCG-vaccine, BCG-brain, or BCG-lung strains at a multiplicity of infection (MOI) of 2. Supernatants were collected and frozen at −80°C for lactate dehydrogenase (LDH) measurements (figure 4c). Statistical analysis for LDH measurement was done using the nonparametric Mann–Whitney U test in GraphPad Prism.

Supernatants were collected and frozen at −80°C for cytokines (figure 5) with ProcartaPlex, Human Th1/Th2/Th9/Th17/Th22/Treg Cytokine Panel (18 plex, eBioscience, San Diego, CA) and CytoTox 96, Non-Radioactive Cytotoxicity Assay (Promega BioSciences, San Luis Obispo, CA), respectively. Cytokine levels from all 3 isolates were compared to untreated cells and within BCG isolates for statistical significance using one-way ANOVA with Bonferroni’s multiple comparisons test in GraphPad Prism.

Cell lysates were prepared for apoptosis measurements (Lysis buffer: Tris-Cl 50 mM, pH 7.4; Nonidet 1%; Triton-X100 1%; 0.15 M NaCl; 2 mM EDTA; Sigma-Aldrich, St. Louis, MO; and Protease Inhibitor Cocktail Set III, EDTA-Free, Merck Millipore, Darmstadt, Germany) and immunoblots performed with PARP and β-actin antibodies (Figure 4A1) (Cell Signaling Technology, Danvers, MA) and horseradish peroxidase-conjugated anti-rabbit (The Jackson Laboratories, West Grove, PA). All statistical analyses on immunoblots used one-way ANOVA with Bonferroni’s test for multiple comparison test in GraphPad Prism software.

## Results

### Microbiology and cell association studies

Strains recovered from the first lung biopsy (BCG-lung), the first scalp sample (BCG-brain), and the BCG-vaccine were tested in this study (table 1). The colony sizes varied among the strains being BCG-vaccine > BCG-brain ≫ BCG-lung (figure 1a). In order to evaluate cells associated with Mycobacteria, PBMCs from healthy controls (n = 6) were infected with mycobacteria for 2 d (multiplicity of infection (MOI) = 2). BCG-brain produced lower number of cell-associated CFUs compared to BCG-vaccine and BCG-lung, with BCG-lung presenting the higher amount of the three strains (p < 0.05 and p < 0.01, respectively; two-tailed paired Student’s *t-*test; figure 1b). Confocal microscopy of infected MDM cells (2 , MOI = 2) confirmed internalization of mycobacteria which were previously transformed with the green fluorescent protein-expressing pMSP12:GFP plasmid (figure 1c). Susceptibility testing showed distinct resistance patterns with higher Rifampin MIC values in BCG-brain (≥1.2 µg/ml) and BCG-lung (0.14 µg/ml) compared to BCG-vaccine (<0.001 µg/ml). Clofazimine MIC values were higher in BCG-lung (3.12 µg/ml) versus BCG-brain (0.78 µg/ml) or BCG-vaccine (0.19 µg/ml) (table 2).

### BCG genomes sequencing and analysis

Phylogenetic analysis revealed a clear clustering of the clinical isolates and BCG-vaccine strain (figure 1d), consistent with the clinical isolates deriving from the vaccine the patient had received. *M. tuberculosis* H37Rv homologs to 13 *Mycobacterium bovis* BCG genes harboring mutations were identified, and sought in the literature for their biological function. Two distinct rifampin-resistance mutations in the *rpoB* gene (Rv0667) were found in BCG-brain (1304A>T, Asp435Val) and BCG-lung (1355 T > C, Leu452Pro). These could be attributed to the differences in rifampin MIC values of ≥1.2 µg/ml (BCG-brain) and 0.14 µg/ml (BCG-lung). A clofazimine resistance mutation (284 T > A, Leu95* premature stop codon) in the Rv0678 (MarR-like) gene found in BCG-lung could be attributed to the higher MIC of 3.12 µg/ml. Mutations in genes affecting lipid biosynthesis (diacylglycerol *O*-acyltransferase) and lipid degradation (*fadA2* and *fadE5*), virulence, and stress response were found (table 3). Lists of high-quality SNPs identified by Illumina read mapping to the gap-free (finished) genome BCG Danish 1331 Belgium [NZ CP039850] are provided in Supplementary file 1. Using a quality cutoff of ≥100 on these VCF files resulted in two non-synonymous SNPs between BCG-vaccine versus the BCG Danish 1331 Belgium [NZ CP039850], which were identified as a hypothetical protein (Rv2267c) and PPE family protein PPE54 (Rv3343c). These identical SNPs were also found in BCG-brain and BCG-lung. Similarly, 10 non-synonymous SNPs were identified between BCG brain and BCG lung, BCG-brain is separated from BCG-vaccine by 7 non-synonymous SNPs, while BCG-lung is separated from BCG-vaccine by 6 non-synonymous SNPs (table 3).

### Mycobacterial lipid analysis

**Figure 3.**
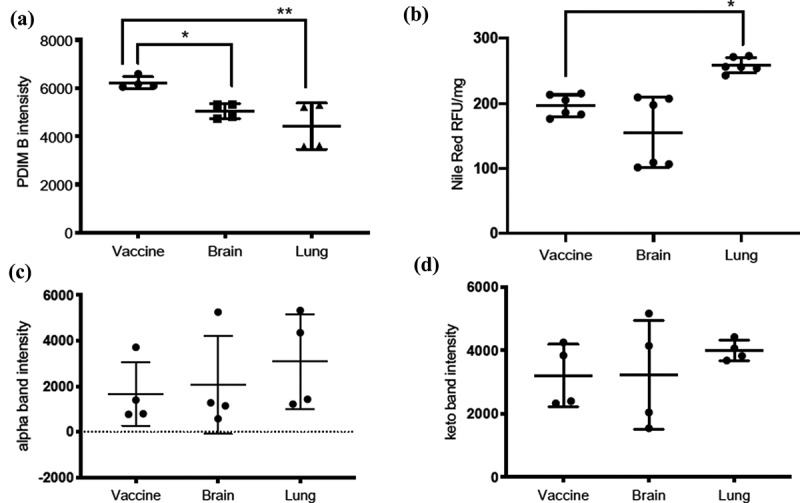
Quantification of PDIMs, neutral lipids, and mycolic acids

Comparison of PDIM TLC profiles revealed dramatically reduced amount of DIMB band by about ~30% in the lung isolate and ~19% in the brain isolate compared to the vaccine isolate (figures 2a & 3a) and were statistically significant with BCG-lung (**p = 0.004) and BCG-brain (*p = 0.043) compared to the vaccine isolate. DIMA or PGL did not show a difference (data not shown).

**(A)** PDIM profiles were quantified using ImageJ and showed a dramatically reduced amount of the DIM B b and of BCG-lung (**p = 0.004) and BCG-brain (*p = 0.043) compared to BCG-vaccine isolate. There were no differences observed in the DIM A band within the three isolates. The graph shows mean ± standard deviation from two independent experiments (*n* = 4). **(B)** Nile red staining of neutral lipids (normalized to the weight of the bacterial pellet) showed the BCG-lung (*p = 0.011) isolate to have a greater amount of neutral lipids compared to the vaccine which was statistically significant while the BCG-brain was lower than the vaccine but statistically insignificant. The graph shows mean ± standard deviation from two independent experiments (*n* = 6). **(C)** The mycolic acid alpha MAME band showed more production in the lung isolate compared the brain and vaccine while **(D)** keto MAME band showed a slight increase in the lung isolate compared to the brain and vaccine, however, these differences were not statistically significant. The values were compared to the BCG-vaccine isolate using one-way ANOVA for multiple comparisons using Bonferroni’s test for statistical significance using the GraphPad Prism software (San Diego, CA). All graphs show mean ± standard deviation from two independent experiments (*n* = 4).

Triacylglycerols (TAGs) TLC profiles comparison revealed ~20% increase in the lung strain and ~6% lower in the brain isolate, compared to BCG-vaccine (figure 2b). Nile red staining of neutral lipids showed; ~ 32% higher lipid content in BCG-lung (*p = 0.011) which was statistically significant and ~21% lower (not significant) lipid content in the brain isolates, compared to the vaccine isolate (figure 3b).

Mycolic acid TLC analysis of MAMEs of BCG-vaccine, brain, and lung isolates revealed the presence of 2 bands for alpha- and keto-mycolic acids (figure 2c), but not methoxy-mycolic acids as seen in *M. tuberculosis* H37Rv [32] or *M. bovis* BCG Tokyo 172 [35]. Interestingly, we noticed an increase in the alpha- and keto-mycolic acids (~2 fold and 25%, respectively) for the lung isolate (figure 3c, 3d) and an increase in the alpha-mycolic acid (~25%) for the brain isolate compared to the vaccine isolate (figure 3c).

### Apoptosis assay

**Figure 4.**
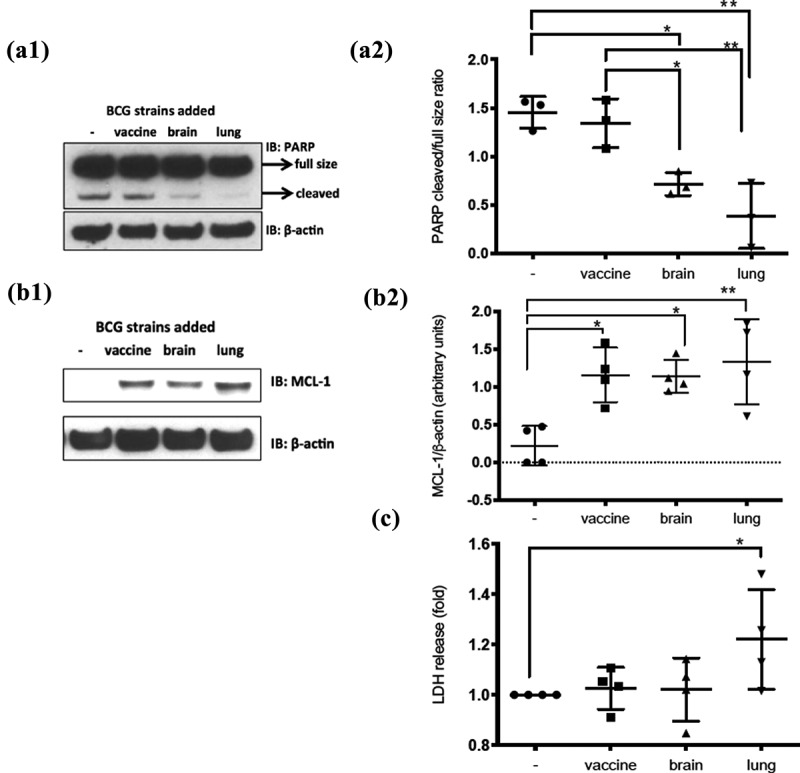
Diminished apoptosis on infected cells by BCG-lung and BCG-brain strains and higher necrosis by infection with BCG-lung measured by LDH-release

Differences in cellular apoptosis were assessed on dTHP-1 cells through caspase-3-mediated cleavage of poly-ADP-ribose polymerase (PARP) [36]. While an elevated baseline level of apoptosis was seen in uninfected cells, a reduction of PARP cleavage was observed upon BCG-lung and BCG-brain infections, more prominent with BCG-lung **p = 0.0015), than BCG-brain (*p = 0.0139) compared to non-infected cells. PARP cleavage was also greater in BCG-brain (*p = 0.0324) and BCG-lung (**p = 0.0030) compared to BCG-vaccine. The significance of the effect of the strain was determined by one-way ANOVA and Bonferroni’s multiple comparisons test (Figure 4A1 and 4A2).

Monocyte-derived macrophage (MDM) cells were used to assess myeloid cell leukemia sequence-1 (MCL-1) protein levels, an anti-apoptotic factor induced by mycobacteria [37]. All three strains upregulated MCL-1, BCG-vaccine (*p = 0.0124) BCG- BCG-brain (*p = 0.0145) and BCG-lung (**p = 0.0038), with a trend to higher MCL-1 levels with BCG-lung (Figure 4B1 and 4B2). When compared to untreated cells, levels were significantly elevated in BCG-lung **p < 0.01 than BCG-vaccine or BCG-brain *p < 0.05.

### Necrosis assay

LDH-release was measured to assess necrosis on mycobacteria-infected dTHP-1 cells [38]. Increased LDH-release was observed on BCG-lung-infected cells, while not among uninfected, BCG-vaccine, or BCG-brain-infected cells (figure 4c) (*p = 0.028, non-parametric Mann–Whitney *U* test).

**(A1)** Cells infected with BCG-brain and BCG-lung strains show markedly reduced apoptosis. dTHP1 cells were not infected or infected with the three different strains of BCG (MOI 2) for 2 d. Protein lysates were subjected to Western blot analysis and proved for PARP and β-actin. Blot shown is representative of three repetitions and **(A2)** Graph is the cumulative densitometric analysis of the ratio of cleaved PARP over the full-size protein. Data, mean ± SD (*n* = 3; *p < 0.05; **p < 0.01; ANOVA). **(B1)** Monocyte-derived macrophages infected at MOI of two with the three strains for 2 d showed higher MCL-1 expression with a trend of higher expression on the lung strain infected cells, blot representative of four repetitions, represented in **(B2)** Graph, is the cumulative densitometric analysis of MCL-1 levels normalized to actin levels (MCL-1/β-actin). Data, mean ± SD (*n* = 4; **p < 0.01; *p < 0.05; ANOVA). **(C)** Necrosis is augmented on dTHP1 cells infected with the BCG-lung strain. The supernatants of dTHP1 cells non-infected or infected with the three strains of BCG at a MOI of 2 were used to quantify the release of LDH from the cells. LDH release is expressed as fold increases when compared to the non-infected condition (arbitrarily defined as “1”). LDH levels were elevated in BCG-lung. Data, mean ± SD (*n* = 4; *p < 0.05; ANOVA)

### Cytokine production

**Figure 5.**
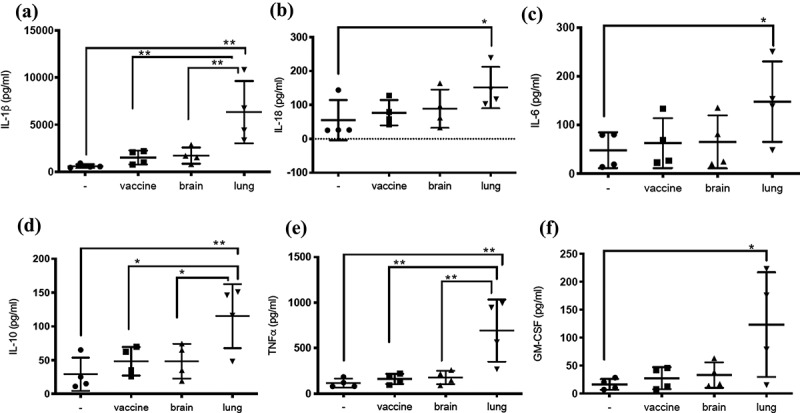
Infection of dTHP1 cells with the BCG-lung strain resulted in a higher secretion of cytokines. The supernatants of dTHP1 cells infected with the three strains of BCG at a MOI of 2 were used to quantify cytokines IL-1β, IL-18, IL-6, IL-10, TNF⍺, and GM-CSF secretion (**A**–**F**). Cytokine levels were compared to untreated cells and within cells infected with BCG isolates. Data, mean ± SD (*n* = 4; *p < 0.05 **p < 0.01; ANOVA)

Cytokine responses were measured in mycobacteria-infected dTHP1 cells (figure 5a-F). Cells infected with BCG-lung produced significantly higher levels of IL-1β (**p = 0.001 BCG-lung, figure 5a), IL-10 (**p = 0.006 BCG-lung, figure 5d), TNF-α (**p = 0.002 BCG-lung, figure 5e) and GM-CSF (*p = 0.030 BCG-lung, figure 5f) when compared to untreated cells. Further IL-1β levels (figure 5a) in BCG-lung infection were significantly higher than BCG-brain (**p = 0.008) and BCG-vaccine (**p = 0.006). Similarly, IL-10 levels (figure 5d) in BCG-lung infection was significantly higher than BCG-brain (*p = 0.033) and BCG-vaccine (*p = 0.0326). TNF-α levels (figure 5e) in BCG-lung infection were significantly higher than BCG-brain (**p = 0.0046) and BCG-vaccine (**p = 0.0036). Statistical significance was determined using one-way ANOVA with Bonferroni’s multiple comparisons test. Cytokine response IL-18 (*p = 0.0279 figure 5b) and IL-6 (*p = 0.033, figure 5c) of BCG-lung infected cells compared to untreated was elevated and was evaluated for statistical significance using one-way ANOVA uncorrected Fisher’s LSD test multiple comparisons test in Graphpad Prism.

**Figure 6.**
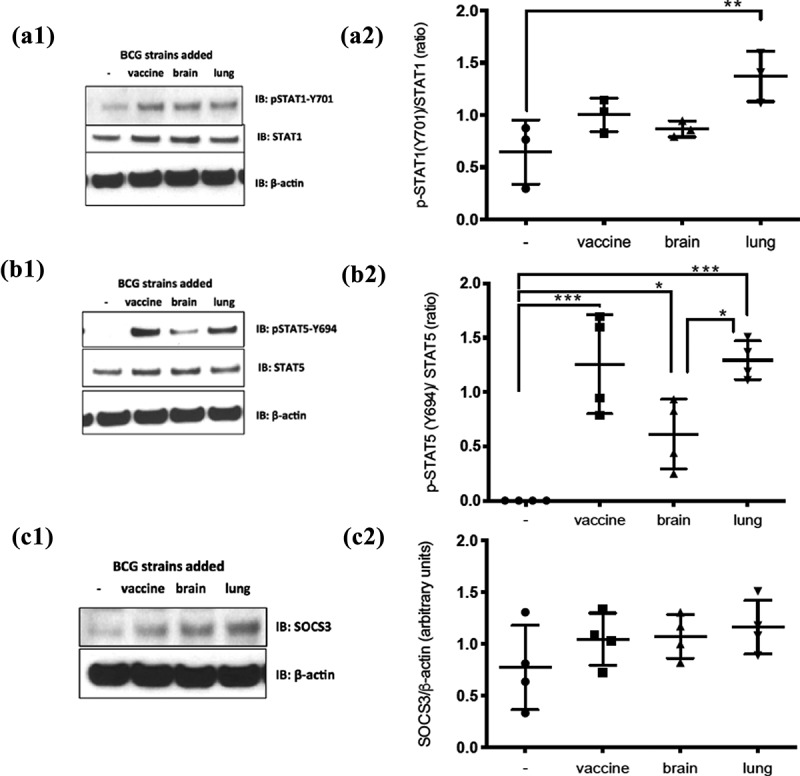
Different alterations in STAT signaling between the lung and brain strains compared to the vaccine strain. Monocyte-derived macrophages infected at MOI of 2 with the three strains for 2 d were probed by western blot for: pSTAT1-Y701, STAT1 total, pSTAT5-Y694, STAT5total, SOCS3, and β-actin (*n* = 3 for STAT1 immunoblots and *n* = 4 for STAT5 and SOCS3 immunoblots). **(A1), (B1),** and **(C1)** representative blots and **(A2), (B2),** and **(C2)** are the cumulative data for the experiments. Data, mean ± SD (*n* ≥ 3; *p < 0.05, **p < 0.01, ***p < 0.001; ANOVA)

### STAT signaling

A higher p-STAT1/STAT1 ratio was observed on MDM cells infected (2 d, MOI = 2) with BCG-lung (**p = 0.0097) when compared to non-infected cells (Figure 6A1 and 6A2). STAT5 phosphorylation was induced by all MDM cells infected with BCG-vaccine, brain, and lung isolates (***p = 0.0002 BCG-vaccine, *p = 0.0363 BCG-brain, ***p = BCG-lung), with lower pSTAT5/STAT5 ratios in BCG-brain (*p = 0.0195) compared to BCG-lung (Figure 6B1 and 6B2). No phosphorylation of STAT3 or STAT4 was detected (data not shown). There was no evidence of a difference in SOCS3 expression in all 3 strains (Figure 6C1 and 6C2).

## Discussion

Two different strains of BCG mycobacteria were isolated from the lung and a central nervous system (brain)-communicating scalp lesion from a patient with partial recessive IFNγR1 deficiency, an immunodeficiency known to cause susceptibility to mycobacteria [12]. Whole-genome sequencing confirmed that BCG-lung and BCG-brain isolates were derived from the original vaccine strain as shown from the phylogenomic tree (figure 1d). However, both isolates were phenotypically and genotypically distinct, as evidenced by their different growth patterns, antibiotic susceptibility, and harboring of particular mutations in genes linked to lipid metabolism, virulence, and antibiotic resistance which were unique to each of the isolates with no evidence of a mixed population. By *in vitro* testing, we explored how these two isolates BCG-brain and BCG-lung independently evolved from BCG-vaccine-derived mycobacteria, show differing antibiotic resistance and immune response in their interactions with human cells. We found that although BCG-brain was more antibiotic resistant, BCG-lung had increased virulence traits, which correlated with the slower improvement in the lung disease clinically.

The growth characteristics differed for the isolates with BCG-lung displaying diminished growth in culture media compared to BCG-vaccine and BCG-brain. Fitness loss for growth *in vitro* was shown to correlate with adaptive survival inside the macrophages [39]. Work in *M. tuberculosis* has uncovered mechanisms to permit the progression of disease, such as changes in lipid metabolism and transcription regulation [4]. Similarly, genomic comparisons among our BCG strains identified mutations in genes affecting lipid metabolism and virulence. BCG-lung displayed a mutation in the Diacylglycerol O-acyltransferase (Rv3087) within the *MymA* operon. The *MymA* operon (Rv3083 to Rv3089) is involved in mycobacterial envelope structure and mycolic acid or fatty acid metabolism, as well as survival in macrophages and guinea pigs [40,41]. Rv3087 was suggested to be required for survival in mice [42] and was shown to be up-regulated inside hypoxic, lipid-loaded THP-1 derived macrophages [43]. Both Rv3087 and Rv3088 contain an HHxxxDG motif common feature of the family of acyltransferases and potentially involved in thioesterification or Claisen type condensation of fatty acids. Further activation of the condensed fatty acids by an acyl-CoA synthase Rv3089 can yield long-chain fatty acids (keto acids) which are precursors of mycolic acids. We hypothesize that the H138R change near the active site of the enzyme may directly or indirectly lead to the observed increase in mycolic acids (keto and alpha MAMEs) in the BCG-lung strain. BCG-lung also displayed a mutation in *hcrA*, a heat-shock protein transcriptional repressor involved in virulence and adaptation [44], while BCG-brain showed a non-synonymous mutation Vso12G in the *fadE5* gene responsible for fatty acid beta-oxidation.

Lipid analysis showed a dramatic reduction in the DIMB band of phthiocerol dimycocerosates (PDIMs) for both BCG-lung and BCG-brain. DIMB is a precursor of DIMA in *M. tuberculosis. M. tuberculosis* mutants deficient in both DIMA and DIMB biosynthesis have been shown to be associated with virulence attenuation of *M. tuberculosis* in mice [45] and more recently it was shown that PDIM contributes to *M. tuberculosis* escape from the phagosome [46]. On the other hand, mutations in the PDIM pathway were associated with a drug-tolerant high-persistence phenotype [47]. Recently, it was suggested that *M. tuberculosis* exploits surface-exposed DIM to destabilize the phagosome and induce phagosomal rupture in infected human macrophages [48]. We hypothesize that BCG-lung and BCG-brain selected an optimal reduced level of PDIMB allowing them to retain virulence and at the same time tolerate environmental stresses posed by antibiotics or the host immune system.

BCG-lung strain was shown to have an increased amount of TAG compared to the vaccine and brain strains. *M tuberculosis* inside the lipid-loaded macrophages imports fatty acids derived from host TAG and incorporates them intact into *M. tuberculosis* TAG [43]. Host lipids may be hydrolyzed by *M. tuberculosis* lipases and the released fatty acids may be imported and re-esterified into *M. tuberculosis* TAG by the action of *M. tuberculosis tgs* gene products. A study by Abdallah et al. [49] describes genomic expression profile differences from several BCG vaccine strains, and it would be interesting to assess expression profiles from the clinical isolates BCG-brain and BCG-lung from the patient.

Acquisition of drug resistance in *M. tuberculosis* is favored by bacterial population increases due to inadequate treatment, compartmentalization of infection to sites with low antibiotic penetration, and impaired host immunity [50]. Our BCG isolates differed in their antibiotic resistance patterns, with the BCG-brain isolate (MIC ≥1.2 µg/ml)) displaying higher Rifampin resistance than BCG-lung (0.14 µg/ml). This was supported in the genomics data with the finding of distinct *rpoB* mutations in BCG-brain and BCG-lung, known to confer high- and low-level Rifampin resistance, respectively, in *M. tuberculosis* genomes [51,52]. We speculate that differences between drug sensitivities to Rifampin could be attributed to the differing SNP mutations in BCG-brain (Asp435Val) versus BCG-lung (Leu452Pro). CSF drug levels were not obtained and it is possible that different antibiotic susceptibility patterns were in part due to diminished penetration of anti-mycobacterial drugs into the central nervous system (CNS). In addition, BCG-lung strain displayed a frameshift mutation with a stop codon mutation (Leu95*) in Rv0678 (MarR-like) encodes a transcriptional repressor of the MmpS5-MmpL5 efflux system [53,54] and was found to be more resistant to clofazimine (MIC µg/ml 3.12) than BCG-brain or BCG-vaccine. Mutations in the Rv0678 causing overexpression of the efflux pump were found to result in cross-resistance to both clofazimine and bedaquiline drugs [53–56].

Differences in the interaction between the BCG isolates and host response were also observed. Pathogenic mycobacteria can use different mechanisms to subvert host cells to perpetuate infection [6,7,9–11] several of which were evaluated in this study. When infecting macrophages, non-virulent mycobacteria trigger apoptosis [7]. Apoptotic-bodies are then phagocytized by antigen-presenting cells, promoting the activation of T cells, which in turn help to clear the infection [7,57]. In contrast, virulent mycobacteria suppress apoptosis of infected cells and favor cell death by necrosis. Necrotic cells release bacilli that can infect other macrophages perpetuating the infection [7]. Previous work by Sly et al. has shown that MCL-1 protein expression, which has antiapoptotic properties, was found to be upregulated during infection with virulent *M. tuberculosis* strain H37Rv [37]. We observed elevated levels of MCL-1 in macrophages infected with BCG isolates compared to uninfected cells in the current study. We found evidence that both clinical isolates BCG-brain and BCG-lung inhibited apoptosis more actively than the BCG-vaccine; and BCG-lung induced more cell necrosis. Recently, an *in vitro* infection of macrophages with *M. tuberculosis* led to an increase in TG or TAGs under conditions of necrosis [58] and support our findings in the current study of increased necrosis and production of TAGs in BCG-lung. These findings suggest that both clinical isolates, and in particular BCG-lung, are more virulent than the BCG-vaccine from which they derive. In addition, quantitative cultures found higher numbers of BCG-lung organisms associated with cells at 2 d post-infection when compared to BCG-brain and BCG-vaccine infectious cultures. Confocal microscopy of cells infected with mycobacteria confirmed the ability of the strains not only to attach but also invade host cells. Experiments assessing intracellular growth in J774 mouse macrophage cell line revealed that both BCG-lung and BCG-vaccine strains grew inside the macrophages with comparable rates. In contrast, while BCG-brain survived inside the macrophages, there was no intracellular growth of this strain compared to the other strains.

In response to mycobacterial infection, impaired host cytokine production can facilitate bacterial growth. On the other hand, too much cytokine secretion can result in tissue damage by excessive inflammatory responses. Therefore, there may be a need for rigorous control of cytokine levels during mycobacterial infection that allows the elimination of mycobacteria without excessive damage to the involved tissues [9,59–63]. The higher pro-inflammatory cytokine levels induced by BCG-lung may have produced increased tissue damage and impaired clinical outcome. Since both BCG-lung and BCG-vaccine showed comparable intracellular growth rate, we hypothesize that initial cell interactions of BCG-lung are responsible for the excessive inflammatory response which is likely driven by differences in cell surface molecules. Increased mycolic acids and in particular alpha-mycolic acids in the lung strain could explain its hyperinflammatory phenotype. Mycolate modification by the cyclopropane synthase *pcaA* (which generates alpha-mycolic acids) was shown to be both necessary and sufficient for proinflammatory activation of macrophages during early infection [64] and was also required for persistence and virulence of *M. tuberculosis* in mice [65]. We were not surprised to see IL-10 increased in BCG-lung, since the vaccine BCG strain is known to be able to upregulate its secretion post-vaccination in humans [66]. Although IL-10 has known anti-inflammatory properties [67], there is evidence pointing to its deleterious effect in mycobacterial infection of humans and mice, for example; inhibiting macrophage phagosome maturation and bacterial clearance in humans [68] and the IL-10^−/−^-deficient mice which cannot produce functional IL-10 are resistant to mycobacterial infection compared to control mice [69]. Therefore, the increase in IL-10 secretion produced by the BCG-lung strain could increase its virulence.

Autophagy, a critical mechanism in the elimination of mycobacteria, was not shown to be different between the three strains by LC3B, SQSTM1, or autophagosome evaluation. These results suggest that the mutations accumulated on the isolates may have spared this pathway. BCG-lung infected cells showed higher STAT1 phosphorylation ratios that could be attributed to cytokine secretion, in particular IL-6 [70]. The lower STAT5 phosphorylation ratio in BCG-brain suggests divergent micro-evolution of a pathogen in different environments within the host. While STAT5b deficient patients do not seem more susceptible to mycobacteria [71], impaired phosphorylation of STAT5 in patients with STAT3 gain of function mutations has been linked to increased susceptibility to mycobacterial disease [72].

The differential features between BCG-brain and BCG-lung and either with BCG-vaccine illustrate a distinct *in vivo* difference in a pathogen in various anatomical sites leading to distinct microbiological and immunological features. This was reflected clinically in that the pulmonary process continued to worsen, therefore stressing the more virulent profile in the BCG-lung strain while the CNS disease stabilized, despite the BCG-brain having a pattern of more resistance to multiple antibiotics. The altered signaling pathways identified in this study can be targeted by drugs other than anti-mycobacterial, that could trigger autophagy or apoptosis in macrophages (e.g. clofazimine) [73,74] or through adjuvant cytokines, such as IFNγ and IFNα that address the partial IFNγR1 deficiency and are known to limit IL-1β secretion [75] and increase apoptosis [76], all mechanisms here proven subverted by the evolved infecting strains. All of these treatment options were used for this patient, and led to clinical improvement; however, the impact of each specific treatment is unclear.

Our findings suggest that *M. bovis* BCG can evolve within the mammalian host. Furthermore, distinct anatomic niches may select pathogens with distinct virulence and antimicrobial resistance patterns. It is unclear whether these changes were the cause or the consequence of the mycobacterial isolation site, or the underlying immunodeficient condition of the host. In addition, although our experiments suggest higher virulence traits for BCG-lung compared to BCG-brain, and both compared to BCG-vaccine. Clinically it is important to recognize that these differential patterns may exist, and that characterization of pathogens from multiple sites may be essential to optimize therapy.

## Supplementary Material

Supplemental Material
