# Supplementary material for: Tissue specific diversification, virulence and immune response to Mycobacterium bovis BCG in a patient with an IFN-γ R1 deficiency
Source: Virulence. 2020 Dec 24;11(1):1656–73. doi: 10.1080/21505594.2020.1848108 (PMC7781554; doi:10.1080/21505594.2020.1848108)
Supplement: Supplemental Material [file KVIR_A_1848108_SM7584.zip › Supplementary_BCG_10-14-2020.docx]

***Supplementary Methods***

***Intracellular growth of BCG strains in infected mouse monocyte macrophage cell line J774***

The mouse monocyte macrophage cell line, J774 cells, was cultured in Dulbecco's Modified Eagle Medium (DMEM, Gibco) supplemented with 10% heat-inactivated FBS (Gibco) in tissue culture flasks. Prior to beginning the assay, the cells were trypsin harvested, plated in 24-well plates at a density of 5×10^5^ cells/well and left overnight at 37ºC to adhere. Cells were then infected with cultured BCG isolates grown in 7H9 to OD_650_=0.2–0.3. Bacterial culture was filtered for clump removal and diluted to 5×10^5^ CFUs/ml. Bacteria were added to the 24-well plates at a MOI =1. The cells were allowed to internalize the bacteria for 24 hours at 37ºC and then were rinsed with fresh medium to remove un-internalized bacteria. The bacteria were allowed to grow internally within the monocytes for 8 days with a wash and replenishment of medium at day 3 before harvesting and enumeration of CFU. For enumeration of viable bacterial numbers on day 0, and internalized numbers on day 1, macrophages were lysed with 0.1% SDS and serial dilutions were plated on 7H11 plates (supplemented with OADC). After 8 days of treatment, all macrophages were lysed (by adding 0.1% SDS) and serial dilutions were plated on 7H11 plates (Supplemented with OADC). The assay was performed in 2 biological replicates each with 4 technical replicates. The mean CFU/ml values were plotted against time for each strain [1] (Supplementary Figure 1). All statistical analyses related to immunoblots were done using one-way ANOVA with Tukey’s multiple comparisons test in GraphPad Prism (San Diego, CA).

***Assays with monocyte-derived macrophages (autophagy)***

Monocyte-derived macrophages (MDM) were differentiated from healthy donor monocytes following negative selection of CD14+ cells from PBMCs (EasySep Human Monocyte Enrichment Kit, STEMCELL Technologies, Seattle, WA) and 5 days incubation with growth media supplemented with 100 ng/ml M-CSF (PeproTech, Rocky Hill, NJ). Following replacement with infection media, GFP-transfected BCG strains (Figure 1D) were added for 2 days (MOI=0.05-2) and cells washed and fixed in paraformaldehyde 4% (USB, Cleveland, OH). Slides were then permeabilized with Triton-X100 1%, blocked with Triton-X100 1% and 10% FBS and incubated with primary antibody LC3B (Abgent, San Diego, CA), goat anti-Rabbit IgG (H+L) secondary antibody, Alexa Fluor 647 conjugate (Thermo Fisher Scientific, Waltham, MA) and then stained with DAPI (Cell Signaling Technology, Danvers, MA). Quantification of nuclei and LC3B-puncta was performed with Imaris 8.1 software spot-counting feature (Supplementary Figure 2C) (Bitplane AG, Zurich, Switzerland).

Immunoblots were performed on protein lysates with LC3B and SQSMT1 antibodies following manufacturers’ recommendations (Supplementary figure 2A1 and 2B1). All statistical analyses related to immunoblots were done using one-way ANOVA with Bonferroni’s multiple comparisons test in GraphPad Prism (San Diego, CA).

***Supplementary Results***

***Evaluation of intracellular growth of mycobacteria***

Intracellular killing of BCG isolates in infected mouse macrophages was evaluated at day 1 and day 8 post-infection. When evaluating CFUs from day 1 and day 8, there was an increase in CFUs for the vaccine (****p=<0.0001, log 1.4) and lung strains (*p=0.0383, log 1.1) on day 8 compared to day 1. In contrast, the brain strain showed no increase in CFUs from day 1 to day 8 (not significant p= >0.999). At day 8 the number of CFUs for vaccine where higher than lung (*p=0.0108) and brain (****p=<0.0001) respectively (Supplementary Figure 1).

***Autophagy evaluation***

Autophagy was studied via LC3B-II expression and Sesquestosome-1 (SQSTM1) degradation on mycobacteria-infected MDM cells (2 days, MOI=2) [2]. All three strains showed trends to lower LC3B-II expression and SQSTM1 degradation compared to control (Supplementary figure 2: A1, A2, B1, B2). No evidence for a difference in LC3B-II expression and SQSTM1 was observed compared to the untreated cells. Confocal microscopy of MDM cells infected with GFP-expressing mycobacteria (2 days, MOI=0.05) was used to determine the number of autophagosomes per cell. As shown in Supplementary figure 2C, cells infected with any of the three strains showed no differences when compared to the control untreated condition.

**References**

1. Kuskovsky R, Lloyd D, Arora K, et al. C4-Phenylthio beta-lactams: Effect of the chirality of the beta-lactam ring on antimicrobial activity. Bioorg Med Chem **2019**; 27:115050.

2. Zheng YT, Shahnazari S, Brech A, Lamark T, Johansen T, Brumell JH. The adaptor protein p62/SQSTM1 targets invading bacteria to the autophagy pathway. J Immunol **2009**; 183:5909-16.

***Supplementary Figures***

**Supplementary Figure 1: Evaluation of intracellular growth of Mycobacteria.**

Intracellular killing of BCG isolates in infected mouse macrophage cell line J774. Murine mouse macrophage cell line J774 cell line (5×10^5^ cells/well) was infected at with averaged inoculums for vaccine (7×10^3^), lung (1.7×10^4^) and brain (1×10^3^) BCG strains respectively. The inoculum was enumerated at the time of infection and internalized bacteria within macrophages on Day 1 and Day 8 post-infection are shown (n=2).

**Supplementary Figure 2: Evaluation of autophagy by infection of the 3 *Mycobacterium bovis* BCG strains.** Infected monocyte-derived macrophages (MDMs) for 2 days at a MOI of 2 were probed by western blot for LC3B, Sesquestosome-1 and β-actin, **(A1)** and **(B1)** representative blots of 4 repetitions on normal donors respectively. **(A2)** and **(B2)** graphs of densitometries of all repetitions. **(C)** Representative fields of MDMs infected at MOI of 0.05 with the 3 GFP-transfected strains, DAPI staining for nucleic acids and Red for LC3B. **(**Data, mean ± SD, n=4).

***Supplementary Files***

**Supplementary File 1:** Synonymous versus non-synonymous SNPs in BCG-vaccine, BCG-brain, BCG-lung versus BCG Danish 1331 Belgium [NZ CP039850].
