# Supplementary figures and images for: Tissue specific diversification, virulence and immune response to Mycobacterium bovis BCG in a patient with an IFN-γ R1 deficiency
Source: Virulence. 2020 Dec 24;11(1):1656–73. doi: 10.1080/21505594.2020.1848108 (PMC7781554; doi:10.1080/21505594.2020.1848108)

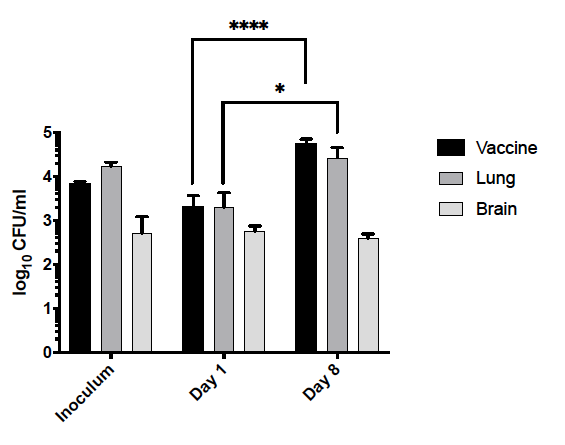

Supplement: Supplemental Material [file KVIR_A_1848108_SM7584.zip › Supplementary_Figure_1_10-14-2020.tiff]

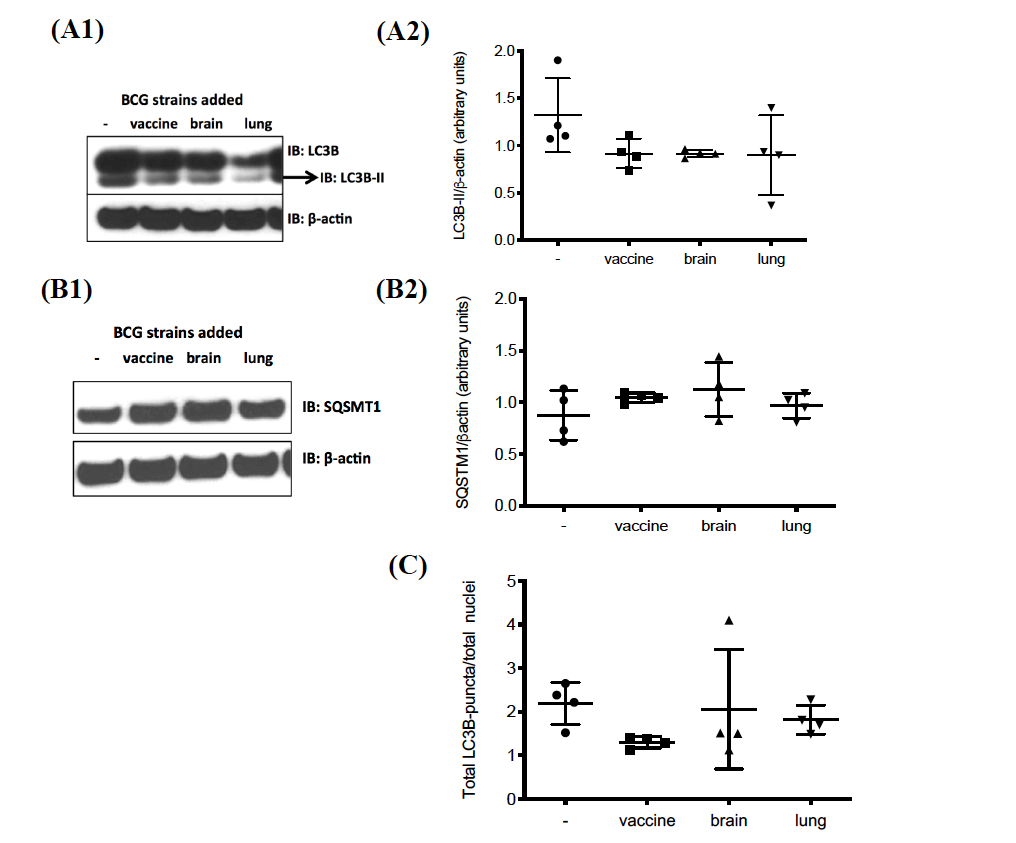

Supplement: Supplemental Material [file KVIR_A_1848108_SM7584.zip › Supplementary_Figure_2A1-A2-B1-B2-C.tiff]
